# Wnt and TGF-β Expression in the Sponge *Amphimedon queenslandica* and the Origin of Metazoan Embryonic Patterning

**DOI:** 10.1371/journal.pone.0001031

**Published:** 2007-10-10

**Authors:** Maja Adamska, Sandie M. Degnan, Kathryn M. Green, Marcin Adamski, Alina Craigie, Claire Larroux, Bernard M. Degnan

**Affiliations:** School of Integrative Biology, The University of Queensland, Brisbane, Queensland, Australia; University of Queensland, Australia

## Abstract

**Background:**

The origin of metazoan development and differentiation was contingent upon the evolution of cell adhesion, communication and cooperation mechanisms. While components of many of the major cell signalling pathways have been identified in a range of sponges (phylum Porifera), their roles in development have not been investigated and remain largely unknown. Here, we take the first steps toward reconstructing the developmental signalling systems used in the last common ancestor to living sponges and eumetazoans by studying the expression of genes encoding Wnt and TGF-β signalling ligands during the embryonic development of a sponge.

**Methodology/Principal Findings:**

Using resources generated in the recent sponge *Amphimedon queenslandica* (Demospongiae) genome project, we have recovered genes encoding Wnt and TGF-β signalling ligands that are critical in patterning metazoan embryos. Both genes are expressed from the earliest stages of *Amphimedon* embryonic development in highly dynamic patterns. At the time when the *Amphimedon* embryos begin to display anterior-posterior polarity, *Wnt* expression becomes localised to the posterior pole and this expression continues until the swimming larva stage. In contrast, *TGF-β* expression is highest at the anterior pole. As in complex animals, sponge *Wnt* and *TGF-β* expression patterns intersect later in development during the patterning of a sub-community of cells that form a simple tissue-like structure, the pigment ring. Throughout development, *Wnt* and *TGF-β* are expressed radially along the anterior-posterior axis.

**Conclusions/Significance:**

We infer from the expression of *Wnt* and *TGF-β* in *Amphimedon* that the ancestor that gave rise to sponges, cnidarians and bilaterians had already evolved the capacity to direct the formation of relatively sophisticated body plans, with axes and tissues. The radially symmetrical expression patterns of *Wnt* and *TGF-β* along the anterior-posterior axis of sponge embryos and larvae suggest that these signalling pathways contributed to establishing axial polarity in the very first metazoans.

## Introduction

Little is known about the morphogenetic complexity of the last common ancestor of modern multicellular animals, but it is generally thought to be an extremely simple organism without a body axis, multiple cell layers and tissues [reviewed in 1]. We can reconstruct this hypothetical animal–the Urmetazoa–by identifying common features in embryonic development of distantly related extant clades, specifically bilaterians, cnidarians, ctenophores and sponges. Among these groups, bilaterians are represented by long-favourite developmental model systems and several hypotheses have been proposed regarding morphogenetic complexity of their last common ancestor–the so-called Urbilateria or protostome-deuterostome ancestor [reviewed in 2]. Recent studies demonstrate surprising similarity between cnidarian and bilaterian gene content and development [3]–[7]. For example, the expression of Wnt genes is associated with blastopore and site of gastrulation in cnidarian and chordate embryos [3], [5], [8], [9]. Even more surprisingly, TGF-β ligands that are involved in determination of the dorsal-ventral axis in bilaterians are also asymmetrically expressed during cnidarian development [6], [7], [10]. Without attempting to homologize the embryonic axes between cnidarians and bilaterians, the existence of two perpendicular embryonic axes, one directed by a Wnt gradient, and the other by a TGF-β gradient in the last common ancestor of living cnidarians and bilaterians appears plausible.

**Figure 1:**
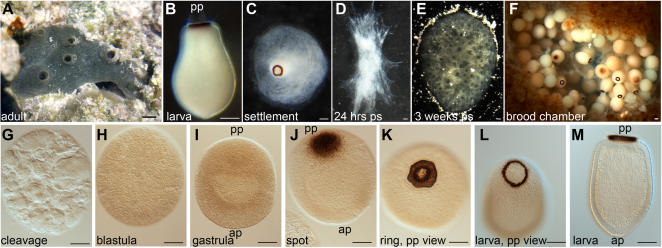
*Amphimedon queenslandica* life cycle and embryonic development. Top panels, live specimens. (A) Adult animal. (B) Swimming larva. (C) Larva undergoing settlement with anterior part flattened on substrate. (D) Postlarva 24 hours post settlement (ps). (E) A three week old juvenile. (F) Sliced brood chamber showing developing embryos of different stages. Bottom panel, benzyl alcohol/benzyl benzoate cleared whole mount developmental stages. (G) After a series of chaotic and asymmetric cell divisions there is a population of unevenly-sized and irregular macromeres and a population of tiny micromeres that are located on the surface and between the macromeres. Micromeres are too small to be seen in this micrograph. (H) A solid blastula is formed. (I) Gastrulation results in a bilayered embryo with the outer layer thicker at the future posterior pole (top); darker pigment cells are distributed throughout the outer layer. (J) Pigment cells migrate through the outer layer towards the posterior pole and coalesce to form a pigment spot. (K–L) The cells of the pigment spot reverse direction and migrate outwards to form a ring. (L, M) Swimming larva with a posterior pigment ring, inner cell mass, ciliated epithelial layer and a subepithelial middle layer. See 21 and 22 for a more detailed description of development and larval cell types. Scale bar is 100 µm on all images except (A) where it is 1 cm. ap–anterior pole, pp–posterior pole.

Until recently, developmental genetic data have not been available from sponges, whose adult body plan has not changed since before the Cambrian explosion [11], [12]. Molecular phylogenies agree that the sponge lineage(s) diverged from the main (eu)metazoan lineage before all other major extant phyla [13]–[19]. Unlike the morphologically more complex eumetazoans, sponges are considered to lack true tissue-level organization and metazoan-specific cell types such as neurons and muscles. Historically, these fundamental differences in the body plans have led to a prevailing view that sponges are living representatives of an evolutionary intermediary between unicellular choanoflagellate protists and the eumetazoans [20]. Indeed, many adult sponges, such as the adult demosponge *Amphimedon queenslandica* (formerly known as *Reniera* sp.; Fig. 1A), have highly plastic body shapes and lack an apparent anterior-posterior (AP) axis of symmetry (Fig. 1A). However, most sponge embryos and larvae have an obvious AP axis with radial symmetry. This similarity to other metazoans is lost at metamorphosis when the growing sponge assumes its sessile body form (Fig 1B–F). Importantly, the formation of a patterned larva with a range of cell types distributed along the AP axis and allocated into different cell layers indicates that sponge embryos must have a requirement for localised signals [21], [22].

The recent sequencing of the genome of the demosponge *Amphimedon queenslandica* by the Joint Genome Institute greatly facilitates reconstruction of the genetic repertoire that was present in the last common ancestor to all contemporary metazoans and reveals the innovations that lead to evolution of the first branches in the animal tree of life [23]–[28]. Amongst these innovations must have been a suite of signalling pathways that allow for communication in a range of multicellular contexts, including cell specification and patterning [22]. The highly conserved Wnt and TGF-β signalling pathways are fundamental to a plethora of developmental processes in bilaterian animals. In addition to specification of the first embryonic axes, these pathways interact to specify cells and to pattern tissues in many morphogenetic contexts, ranging from the formation of embryonic organizers [29]–[32], vertebrate skeleton [33] and the development of limbs in *Drosophila* and other bilaterians [34]–[36]. The primacy of Wnt and TGF-β pathways in intercellular communication and cell fate diversification suggests that their evolution may have been concomitant with the origin of multicellularity [22], [37]. Here we address this issue by investigating the expression of *Wnt* and *TGF-β* genes during embryonic development in *Amphimedon queenslandica*. The asymmetrical expression of both genes in *Amphimedon* embryos indicates that sponges, and hence also the last common ancestor to living metazoans, utilized these two signalling pathways in embryonic patterning.

## Results

### *Amphimedon* embryogenesis

*Amphimedon queenslandica* embryos develop in brood chambers, with different developmental stages found together in one chamber (Fig. 1F). Early cleavage stages are milky-white and are found mainly at the edges of the brood chamber (Fig. 1F–G). At this time, cell divisions appear highly asymmetric and asynchronous, and the embryos are composed of irregularly shaped macromeres of various sizes with small micromeres interspersed between them (Fig. 1G). A solid blastula, with more uniformly sized cells is formed at the end of this process, and it does not display any morphological asymmetry (Fig. 1H). Different cell populations present in the blastula sort themselves into layers in a process that we consider to be gastrulation [21]. At the end of gastrulation, the outer layer is composed of smaller micromeres including pigment cells that give embryos a beige colour; bigger macromeres are present in the inner cell mass (Fig. 1I). While no asymmetry can be observed in live embryos, cleared beige coloured embryos reveal striking anterior-posterior asymmetry, with the outer layer significantly thicker at the posterior pole (Fig. 1I). Pigment cells initially distributed throughout the outer layer soon begin migration towards the posterior pole (Fig. 1J), where they coalesce into a spot, and then begin outwards migration resulting in formation of a narrow pigment ring (Fig. 1 J–L). At the same time, multiple cell types migrate along the anterior-posterior axis to yield a highly patterned larva that consists of multiple cell layers, each of which contains a number of distinct cell types [21, Fig. 1L–M].

### Wnt and TGF-β ligands are present in *Amphimedon*

We isolated *Wnt* and *TGF-β* genes from *Amphimedon* using a combination of EST and genome trace searches and RACE cloning. The deduced *Amphimedon* Wnt protein contains a signal peptide and 24 conserved cysteines characteristic for this family [38], [39] (Fig. S1), although phylogenetic analyses can not confidently assign this sponge Wnt into a recognised eumetazoan sub-family (Fig. S2) [39].

Phylogenetic analysis of the predicted *Amphimedon* TGF-*β* signalling domain places it close to the GDNF subfamily of TGF-β molecules (Fig. S3). Of the 7 cysteines that are conserved in the signalling domain of TGF-*β* superfamily, 6 are present in the *Amphimedon* sequence (Fig. S4). Cysteine 4 is missing, as it is the case in mouse GDF3, GDF9 and BMP15 proteins [40]. Since this particular cysteine is responsible for intermolecular disulfide bond formation in a mature dimer, it appears that the *Amphimedon* TGF-*β* can act as a monomer or forms a non-covalent dimer [40]. The preprotein contains a signal peptide and a conserved proteolytic cleavage site RTRRS, lending further support to its assignment to this signalling protein family (Fig. S5) [41].

### Expression of *Wnt* and *TGF-β* genes during *Amphimedon* development

**Figure 2:**
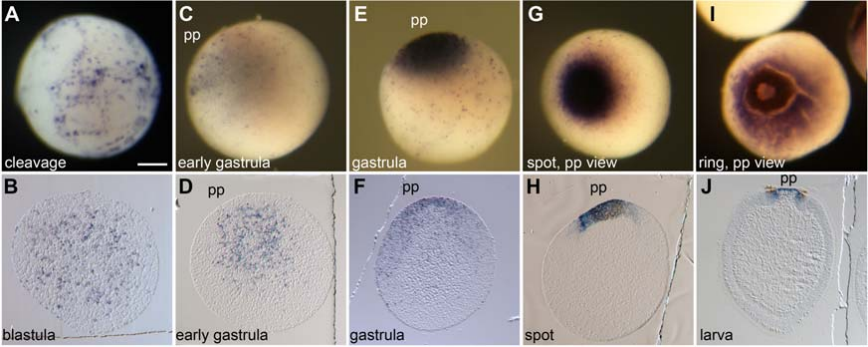
Expression of *Wnt* in *Amphimedon* embryos. Top panel, whole mount in situ hybridizations; bottom panel, sectioned in situ hybridisations. (A) During cleavage, *Wnt* is expressed in micromeres distributed throughout the early embryo. (B) At the blastula stage, Wnt-expressing cells are predominantly localised interiorly. (C, D) Asymmetric localisation of *Wnt*-expressing cells occurs early in gastrulation, initially inside of the embryo. (E, F) As the gastrulation progresses, *Wnt*-positive cells are evident in the outer layer in the posterior pole. (G, H) At the spot stage, *Wnt* expression overlaps with the pigment spot and extends beyond it. (I) This expression pattern is maintained during ring formation. (J) Expression of *Wnt* continues in the posterior pole in swimming larvae. Scale bar is 100 µm. ap, anterior pole; pp, posterior pole.

We studied the expression of the identified genes using whole mount in situ hybridization on *Amphimedon* embryos and larvae. *Amphimedon Wnt* gene is expressed from the early stages of development (Fig. 2). There is no evidence that *Wnt* transcripts are maternally deposited in oocytes or eggs. During cleavage, the *Amphimedon* embryo consists of large macromeres of varying size and shape surrounded by many tiny micromeres [Fig. 1G; 21]. *Wnt* transcripts first can be detected in very small micromeres that are uniformly distributed throughout the embryo and interspersed between the macromeres (Fig. 2A). At the next recognizable stage of development, the blastula stage, the embryo consists of more evenly sized cells [Fig. 1H; 21]. At this stage, *Wnt*-expressing cells are enriched in the inner part of the embryo (Fig 2B). Before any morphological asymmetry in the embryo can be detected by cytological indicators [21], [22, unpublished], *Wnt*-expressing cells become restricted to the inner cell mass on one side of the embryo (Fig. 2C, D). We have called this stage early gastrulation based on these localized *Wnt* expression patterns. As gastrulation progresses and separation of outer and inner layer becomes apparent, the *Wnt-*expressing cells become confined to the outer layer at the posterior pole (Fig 2E, F). The posterior pole is relative to larval swimming direction and where the pigment cells will eventually coalesce and form a ring [Fig. 1J]–[M, 21, 22]. The *Wnt* expression domain overlaps with the pigment spot and ring (Fig. 2G–I) and this expression continues within the pigment ring in the free swimming larva (Fig. 2J).

**Figure 3:**
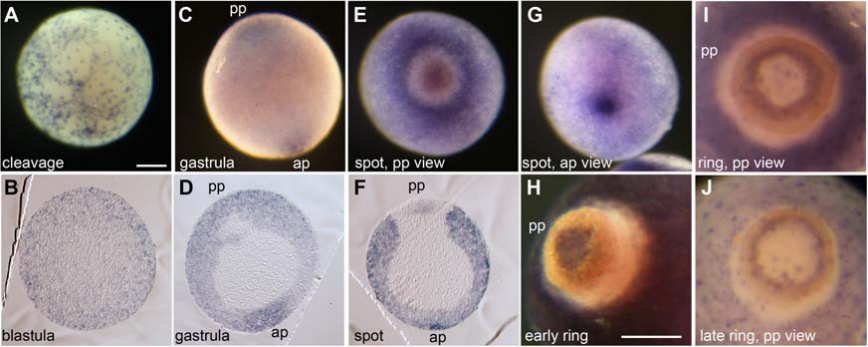
Expression of *TGF-β* in *Amphimedon* embryos. (A) *TGF-β* expressing micromeres are distributed uniformly during cleavage. (B) At the blastula stage, *TGF-β-*positive cells are more prominent in the outer layer. (C, D) During gastrulation, *TGF-β* expression is restricted to the outer layer, with two stronger domains at the anterior and posterior poles. (E, F) As the pigment cells migrate to the posterior pole, *TGF-β* expression disappears from the posterior pole leaving the area just outside the pigment spot clearly devoid of *TGF-β* transcripts. A weak *TGF-β* expression domain persists in the very center of the spot. (F, G) The anterior pole expression remains strong at the spot and ring stages. (H) As the pigment cells begin their outward migration, *TGF-β* expression is strong inside of the forming ring and in the outer layer except of the pigment ring itself and area just outside of it. (I, J) In the later ring, *TGF-β* expression clears from the center of the ring, but persists in the inner rim of the ring. (J) The embryonic expression gradually fades at late ring stages, and expression in some cells of the follicle layer on the surface of the embryo becomes more apparent. Scale bar, 100 µm.

Similar to *Wnt* expression, *TGF-β* expression is first detectable during cleavage stage in small micromeres, which do not appear cytologically different from the Wnt expressing cells (Fig. 3A). However, at the blastula stage *TGF-β*-expressing cells are more prominent in the outer region (Fig. 3B), while Wnt expressing cells are enriched in the inner region (Fig. 2A), indicating that these are largely different populations of cells. During gastrulation, *TGF-β* expression becomes restricted to the outer layer, with stronger domains of expression at anterior and posterior poles of the embryo (Fig 3C, D). Thus, at the gastrula stage, the posterior domain of *TGF-β*-expression overlaps with *Wnt*-expression at the posterior pole. At the pigment spot stage (Fig. 1J, 3E–F), *TGF-β* expression is maintained in the centre of the spot and throughout the outer layer, except in pigment cells making the outer portion of the spot and the region immediately adjacent to the spot (Fig. 3E, F). The anterior pole domain of *TGF-β* expression is particularly prominent at the spot stage (Fig 3F, G). The anterior region of the larva consists of a different cell type than the majority of the outer layer [21]. As the pigment cells begin to move concentrically away from the posterior pole to form the pigment ring [Fig. 1K], [21, 22], *TGF-β* is expressed inside of the ring (Fig. 3H). As pigment ring formation progresses, *TGF-β* expression is not longer detected in the centre of the ring, but delineates the inner rim of the ring (Fig. 3I, J). During the ring formation stages, *TGF-β* expression continues throughout the outer layer except of the narrow band of cells just outside of the ring (Fig. 3I, J). *TGF-β* expression in the embryo gradually decreases, and the transcripts are not detected in the swimming larvae (not shown).

## Discussion

We have identified a *Wnt* and a *TGF-β* gene from the *Amphimedon* genome, extending the origin of these important gene families to before the divergence of sponge and eumetazoan lineages. While other studies on sponges have detected components of Wnt and TGF-β signalling pathways in sponges [37], [42]–[44], this is the first to show that these pathways are expressed during sponge embryogenesis. Significantly, we demonstrate that Wnt and TGF-β ligands are expressed during *Amphimedon* embryogenesis in complex and localized patterns.

The early expression of *Wnt* and *TGF-β* in *Amphimedon* embryos is compatible with a role in establishing axial polarity. The localization of *Wnt*-expressing cells to the future posterior pole is the earliest morphogenetic asymmetry detected in *Amphimedon* (Fig. 2C, D), occurring prior to the initial cell sorting event that creates the embryonic cell layers. We can not discern if the initial localized expression of *Wnt* is in the same cells that express *Wnt* later in development because of a lack of cell lineage data. Regardless, it is evident that *Wnt*-expressing cells are restricted early to the posterior pole and *Wnt* transcripts localize to this pole continuously through to the larval stage. The migration of pigment cells towards *Wnt*-expressing cells at the posterior pole is indicative of the existence of differential signals along the AP axis and is compatible with Wnt and/or TGF-β contributing to the establishment of this axis. Also migrating with the pigment cells along the outside of the embryo are sclerocytes-cells responsible for the synthesis of siliceous spicules [21], [23]. Upon reaching the posterior end of the larva, the sclerocytes appear to ingress into the inner cell mass [21]. The movements of pigment cells and sclerocytes are consistent with a role for these metazoan-specific ligands interacting to establish axial polarity in sponge embryos in a manner akin to that observed in other metazoans [1], [30], [45], [46]. For example, the vertebrate organizer is localized by interactions between Wnt and TGF-β signalling pathways [29]–[31], with TCF and SMAD, as respective effectors of these pathways, cooperating to regulate gene expression [31]. In both *Amphimedon* and the cnidarian *Nematostella*, *Wnt* expression is restricted to the posterior ends of the larva [3], [5], although mechanisms of gastrulation are different and there is limited evidence for these poles being homologous.

The intersecting expression of *Wnt* and *TGF-β* at the posterior end of the larva later in development also is compatible with these signalling pathways regulating the formation of the pigment ring [i.e. tissue morphogenesis, 22]. A zone of intersecting *Wnt* and *TGF-β* expression occurs anterior of the leading edge of the concentric front of migrating pigment cells (compare Fig. 2I and 3 H–J), and may be providing positional information [47] in manner similar to that observed during the formation of limbs in *Drosophila* [34] and the head organizer in cnidarians [48].

**Figure 4:**
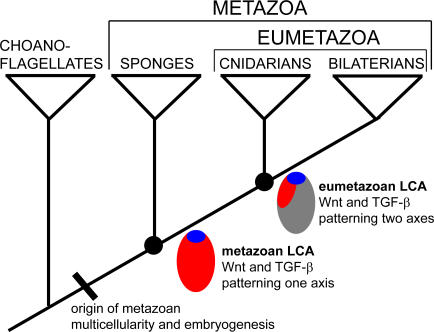
Evolution of Wnt and TGF-β gradients in early metazoans. Metazoan tree of life showing relationships between sponges, cnidarians and bilaterians, and a closely-related sister taxon (choanoflagellates). Major evolutionary transitions in Wnt (blue) and TGF-β (red) gradients during embryonic development. *Wnt* and *TGF-β* genes arose early in metazoan evolution, after the choanoflagellate lineage diverged from the metazoan ancestor. These genes encoded ligands that originally formed gradients along one embryonic axis, as observed in modern sponges. The TGF-*β* gradient shifted to a perpendicular position in relation to the Wnt gradient in the lineage leading to the last common ancestor of contemporary eumetazoans, contribution to evolution of a second body axis.

Our results suggest that sponge embryos are patterned by signalling mechanisms strikingly similar to those controlling cell specification and patterning in bilaterians and cnidarians. These signalling systems evolved and interacted early in metazoan evolution prior to the first cladogenic events that predate the Cambrian explosion (Fig. 4). Wnt and TGF-β signalling pathways appear to have acted combinatorially to specify and pattern cells in the last common ancestor to all extant metazoans (Fig. 4). In addition, a hedgehog-like cell surface signal–Hedgling–is expressed in overlapping patterns with Wnt and TGF-β during ring formation [25]. The developmental expression of these signalling systems, along with that of many metazoan-specific transcription factor families [23], indicates that the last common ancestor to all living metazoans already possessed the regulatory capacity to form complex body plans, using the same molecular components as animals living over 550 million years later [49]. The evolution of this canonical zootypic network may have been the necessary precursor for the diversification of all contemporary metazoan body plans. The differential expansion and elaboration of this network in the eumetazoan lineage, compared to the sponge lineage, provided the foundation for the extensive body plan diversification seen in this clade, including possibly the origin of a second body axis. Along with the diversification signalling pathways in eumetazoans was the origination of Hox genes [24] and expansion of a range of developmental transcription factor gene families [23], [28, unpublished], which enabled further elaboration of ancestral gene regulatory networks.

## Materials and Methods

### Isolation of *Amphimedon Wnt* and *TGF-β* cDNAs

Genomic traces and ESTs were generated as part a collaborative genome project with the Joint Genome Institute and are publicly available (http://www.ncbi.nlm.nih.gov/Traces). The 3′ end of Wnt and TGF−β genes were identified in EST and genome trace archives, respectively, based on similarity to vertebrate sequences. The 5′ part of these genes was cloned by means of 5′ RACE using BD Smart Kit (ClonTech) and gene specific primers (TGF−β: TCTAATCCGAGTAAGAGTATACACAGCTGC; Wnt: TCGCAAAGTTCTGCTGGCAG) and embryonic RNA as template. In case of TGF−β, the RACE primer encompassed the stop codon and several independent clones constituted the entire ORF. In case of Wnt, the complete coding sequence was confirmed by RT-PCR of embryonic RNA (using primers: CATTGACGTACAGCTACAAAG and CATGTTCTTGTGAATAGACTC).

### Whole mount in situ hybridization

Whole mount in situ hybridizations were performed as described in [36] using complete coding sequences cloned into pGEMT vector (Promega) as templates for probe synthesis. Embryos were photographed whole mount and then subsequently processed for sections. Samples were dehydrated in ethanol and infiltrated with Epon 812 resin in a BioWave microwave oven (Pelco) before polymerisation overnight at 60C in a conventional oven. Sections were cut at 5 um on an Ultracut T ultramicrotome (Leica) and mounted in Histomount.

### Phylogenetic analyses

Phylogenetic analyses of Wnt and TGF-β sequences were performed for the purpose of assigning orthology. Detailed description of the methods used is included in Supplement S1.

## Supporting Information

Supplement S1 (0.04 MB DOC)

Figure S1 (0.13 MB DOC)

Figure S2 (0.04 MB DOC)

Figure S3 (0.03 MB DOC)

Figure S4 (0.04 MB DOC)

Figure S5 (0.04 MB DOC)
