# Supplementary material for: Wnt and TGF-β Expression in the Sponge Amphimedon queenslandica and the Origin of Metazoan Embryonic Patterning
Source: PLoS One. 2007 Oct 10;2(10):e1031. doi: 10.1371/journal.pone.0001031 (PMC2000352; doi:10.1371/journal.pone.0001031)
Supplement: Supplement S1 — (0.04 MB DOC) [file pone.0001031.s001.doc]

## Supporting Information

**Wnt and TGF-β expression in the sponge *Amphimedon queenslandica* and the origin of metazoan embryonic patterning**

## Maja Adamska, Sandie M. Degnan, Kathryn M. Green, Marcin Adamski, Alina Craigie, Claire Larroux and Bernard M. Degnan

## This file includes:

## Materials and Methods

**Figure Legends S1 to S5**

**References**

## Materials and Methods

## Phylogenetic data analysis. Phylogenetic analyses of Wnt and TGF-β sequences were performed for the purpose of assigning orthology. Protein sequences obtained from NCBI and Ensembl databases were aligned in 3D-Coffee (Wnt) (Armougom, 2006) and Clustal X (TGF-β) (Thompson et al., 1997), with manual adjustments made by eye. Consistency of phylogenetic signal in the data was tested via three common methods of phylogenetic reconstruction, each with different assumptions: maximum parsimony (MP) using PAUP* 4.0b (Swofford, 2002), maximum likelihood (ML) using PROML from the PHYLIP package (Felsenstein, 1989), and Bayesian monte carlo markov chain (MCMC) methods using MrBayes (Ronquist and Huelsenbeck, 2001). For the Wnt protein family (444 amino acids), 109 characters were constant and only 50 of the 335 variable characters were parsimony-informative. For the TGF-β protein family (115 amino acids), 13 characters were constant and only 8 of the 102 variable characters were parsimony-informative. Parsimony analyses used heuristic searches each with 500 random addition sequence starting trees and tree bisection-reconnection branch-swapping. Clade support was assessed by bootstrapping (1,000 replicates). For ML, the most appropriate model of amino acid substitution for these data was assessed from competing models using the Akaike Information Criterion implemented in ProtTest (Abascal et al., 2005). The best model for both data sets was identified as the WAG model (Whelan and Goldman, 2001) with a proportion of invariable sites and a gamma distribution (WAG + I + G). ML clade support was estimated by bootstrapping (100 replicates plus 10 times sequence order randomization for each replica). Bayesian analyses coupled with MCMC simulations were run under the same model. Four Markov chains, one of which was heated, were run simultaneously for 500 000, 1 million, or 2 million generations and trees were sampled every 100 generations only after posterior likelihoods reached stationarity.

## Supporting Figure Legends

**Figure S1** Alignment of derived metazoan Wnt protein sequences. The *Amphimedon queenslandica* sequence is denoted by the Amq prefix. Black dots, conserved cysteine sites.

**Figure S2** Maximum Parsimony (A) and Bayesian inference (B) midpoint-rooted trees of the Wnt proteins. The analysis is based on part of alignment S1 containing all conserved cysteines (position 93-536). (A) Clade support values are indicated to the right of nodes in the following order: Maximum Parsimony (black), Maximum Likelihood (red), Bayesian (blue). -- indicates MP or ML support < 50%. (B) Bayesian posterior probabilities are indicated to the right of nodes. Amq: *Amphimedon*, mm: mouse (*Mus musculus*), bf: Amphioxus (*Branchiostoma floridae*), hv: hydra (*Hydra vulgaris*), nv: sea anemone (*Nematostella vectensis*), pd: polychaete (*Plathynereis dumerlii*), pv: common limpet (*Patella vulgata*), at: house spider (*Achaearanea tepidariorum*).

The orthology of the AmqWnt is not well resolved; its position in our trees is labile. In addition, relationships among clades are not strongly supported, and all collapse to a polytomy in a 50% majority rule consensus topology. The MP analysis resulted in 144 equally parsimonious trees that did not differ significantly from each other; the ‘best’ (shortest) of these is depicted in Figure S2A. The ML analysis (not shown) returned a similar tree. The Bayesian tree (Fig. S2B) has a significantly different topology with respect to the position of AmqWnt, and more internal nodes were resolved. In both the MP and the ML analyses, the orthology of AmqWnt is ambiguous; it has tenuous associations with the putative Wnt7 and Wnt8 clades, but with very low support (< 50%). By comparison, the Bayesian analyses give moderate support (posterior probability 94) to the orthology of AmqWnt with the Wnt16 clade. Hypothesis testing indicated that MP trees with AmqWnt constrained to the Wnt16 clade were significantly longer (at P<0.05) than the option presented in Fig. S2A, according to the Kishino-Hasegawa test implemented in PAUP (see Goldman et al., 2000). We thus conclude that our data could not unambiguously resolve the orthology of AmqWnt.

**Figure S3** Maximum parsimony midpoint-rooted tree of the conserved signaling domain of TGF-β family of proteins. Clade support values are indicated to the right of nodes in the following order: Maximum Parsimony (black), Maximum Likelihood (red), Bayesian (blue). -- indicates value lower than 50%. Amq: *Amphimedon*, mm: mouse (*Mus musculus*), bf: Amphioxus (*Branchiostoma floridae*), dr: zebrafish (*Danio rerio*), xl: clawed frog (*Xenopus laevis*), nv: sea anemone (*Nematostella vectensis*), am: coral (*Acropora millepora*), gg: chicken (*Gallus gallus*).

The orthology of the AmqTGFβ is less ambiguous than that of the AmqWnt. In all three analyses, it consistently is associated with the GDNF subfamily of TGF-β genes, albeit with only moderate support (MP 88, ML <50, Bayesian 97). We conclude that AmqTGFβ may be a divergent member GDNF subfamily of TGF-β genes.

**Figure S4** Alignment of amino acid sequences of the signaling domain of TGF-β protein family. Black dots, conserved cysteines of the signaling domain; white dot, cysteines present in the majority of TGF-β peptides.

**Figure S5** Alignment of complete pre-protein sequences of *Amphimedon* TGF-β and mouse BMP2. Black dots, conserved cysteines of the signaling domain; black triangle, predicted cleavage site (RXXR).

# References

Abascal, F., Zardoya, R., Posada, D. ProtTest: selection of best-fit models of protein evolution. *Bioinformatics* **21**, 2104-2105 (2005).

Armougom F, Moretti S, Poirot O, Audic S, Dumas P, Schaeli B, Keduas V, Notredame C. Expresso: automatic incorporation of structural information in multiple sequence alignments using 3D-Coffee. *Nucleic Acids Res.* **34** (Web Server issue), W604-8 (2006).

Felsenstein, J. PHYLIP: Phylogeny Inference Package (Version 3.2). *Cladistics* **5**, 164-166 (1989).

Goldman, N., Anderson, J.P., Rodrigo, A.G. Likelihood-based tests of topologies in phylogenetics. *Systematic Biology* **49**, 652-670 (2000).

Hinman, V. F. & Degnan, B. M. Retinoic acid perturbs Otx gene expression in the ascidian pharynx. Dev. Genes Evol. 210, 129-139 (2000).

Ronquist, F., Huelsenbeck, J.P. MrBayes 3: Bayesian Phylogenetic Inference Under Mixed Models. http:/morphbank.ebc.uu.se/mrbayes (2001).

Swofford, D.L., PAUP*. Phylogenetic Analysis Using Parsimony (*and other methods). Version 4. Sinauer Associates, Sunderland, Massachusetts. (2002).

Thompson, J.D., Gibson, T.J., Plewniak, F., Jeanmougin, F., Higgens, D.G. The ClustalX windows interface: flexible strategies for multiple sequence alignment aided by quality analysis tools. *Nucleic Acids Research* **25**, 4876-4882 (1997).

Whelan, S., Goldman, N. A general empirical model of protein evolution derived from multiple protein families using a maximum-likelihood approach. *Molecular Biology Evolution* **18**, 691-699 (2001).
