# Supplementary material for: Wnt and TGF-β Expression in the Sponge Amphimedon queenslandica and the Origin of Metazoan Embryonic Patterning
Source: PLoS One. 2007 Oct 10;2(10):e1031. doi: 10.1371/journal.pone.0001031 (PMC2000352; doi:10.1371/journal.pone.0001031)
Supplement: Figure S1 — (0.13 MB DOC) [file pone.0001031.s002.doc]

**Figure S1 Wnt sequence alignment**

* 20 * 40 * 60 * 80 * 100
HvWnt : M-----------------------------GTTRYKE-TLLCFLLIFM-----ETQAQLWMALG--TQT--SAIESRP-RSSIN----KN-LCRALY--L : 53
bfWnt3 : M-----------------------------DSVRCQV-SVLLLFVIWSQTADVGMGSPGWWYLAVGPQF--SSLAASN-AGGQGRR-WPV-VCSSIPG-L : 64
mmWnt3 : M-----------------------------EPH---L-LGLLLGLLLSGTR-VLAGYPIWWSLALGQQY--TSLASQ-----------PL-LCGSIPG-L : 51
mmWnt3a : M-----------------------------AP------LGYLL-VLCSLKQ-ALGSYPIWWSLAVGPQY--SSLSTQ-----------PI-LCASIPG-L : 48
NvWnt3 : M-----------------------------RVIITA--------IVPLIS-LLVSSEAVWWTLGTQLSV--SSDPAK----------KSM-LCYVTRQ-F : 48
NvWnt10 : M---------------------------------DFRG-FVKVLLIFFQIH-SK--GFGYG---------ILKTSIPFE--DPVIN-SNT-VCKNTPS-L : 49
bfWnt10 : ---------------------------------------------------------------------------------------------------- : -
mmWnt10a : MGS----------AHPRPWLRLPQGP----QPRPEFWA-LLFFLLLLAAAV-PR--SAPND---------ILGLRLPPE---PVLN-ANT-VCLTLPG-L : 67
mmWnt10b : M-----------------------LE----EPRSRPPP-LGLAGLLFLALF-SR--ALSNE---------ILGLKLPGE---PPLT-ANT-VCLTLSG-L : 54
NvWnt11 : M-----------------------------YSLFGLFV-VLCLSFW---SP-VV--TIRWLGIRHTSQ-------------H--TW-DKK-DCNRIHG-F : 46
bfWnt11 : M-----------------------------DFTPALWV-ILLVMFI--DGR-SS--AIKWLSIADVGK-------------NLSWN-KTS-SCRKVSG-F : 49
mmWnt11 : M-----------------------------RARPQVCE-ALLFALALHTGV-CY--GIKWLALSKTPA-------------ALALN-QTQ-HCKQLEG-L : 51
mmWnt9a : MLD----------GS---------------LLARWLAA-AFGLTLLLA-AL-RP--SAAYFGLTGSEPLTILPLTLETE--AAAQA-HYK-ACDRLK--L : 64
mmWnt9b : M-----------------------------RPAPALAL-AALCLLVLP-AA-AA--AAAYFGLTGREVLTPFPGLGTAAAPAQAGA-HLK-QCDLLK--L : 62
NvWnt8 : L--------------------------------------------------------------------------------------------------- : 1
NvWnt8b : M-P-----------------------------------------------C-QG--QSRK-KM-AV-------------------------------L-K : 15
bfWnt8a : M-F----------AQ---------------LLWSLSVL-LA-----ALSGT-AD--SNGW-TLQSN-------------------------------F-L : 32
mmWnt8a : M------------GH---------------LLMLWVAA-GM-----CYPA--LG--ASAW-SV-NN-------------------------------F-L : 29
mmWnt8b : M-----------------------------FLMKPVCV-LL-----VTCVL-HR--SHAW-SV-NN-------------------------------F-L : 28
NvWnt7a : -----------------------------------------ISFSVQ-NAL-ET--GSKKLFVD-----------------SVAIP-PSI-ICTRIQP-L : 35
NvWnt7b : MA-----------KW---------------CFATFIRL-TLISFSVQ-NAL-ET--GSKKLFVD-----------------SVAIP-PSI-ICTRIQP-L : 49
AmqWnt : MA-----------FT---------------SLATAVCL-LMVFNGCL-ASW-WS--LGVYNDLS-----------------ERVVD-S-T-PCESLTNIL : 49
NvWnt16 : M------------LYRTVRLRWPF---------------SLVWWT----VL-IVDANASWWWMGISASG--------S---ISGNS-VSP-SCSRVPG-L : 54
atWnt16 : M-------------LSKTTTFIFL---------------CSFLPLA----------WTNWMYLGMVGPH--HSVT-PP---EMASE-QSS-MCSSVPG-L : 53
mmWnt16 : M-------------DRAALLALPS---------------LCALWAAVLSLL-PCGTQGNWMWLGIAS---------------FGVP-EKL-GCADLP--L : 52
NvWnt2 : M-----------------------------APAKARLG--LLV-LLILLYF-PRKTESHWWFISQ----------------VFALG-AKV-MCNSITG-L : 48
mmWnt2 : M-----------------NVPLG--------------GIWLWL-PLLLTWL-TPEVSSSWWYMRA----------------T-GGS-SRV-MCDNVPG-L : 47
mmWnt2b : MLKLQGEDEAAQLAPRRARVPVPRPTAPDVSPSSARLGLACLL-LLLLLTL-PARVDTSWWYIGA----------------L---G-ARV-ICDNIPG-L : 76
bfWnt7a : ---------------------------------------------------------------------------------------------------- : -
mmWnt7a : M-----------------------------TRKARRCLGHLFL-SLGIVYL-RIGG------FSS----------------VVALG-ASI-ICNKIPG-L : 44
mmWnt7b : M-----------------------------HRNFRKWIFYVFL-CFGVLYV-KLGA------LSS----------------VVALG-ANI-ICNKIPG-L : 44
bfWnt7b : M-----------------------------WPQDYNRYLGAVL-CVAI--L-QLGS------LST----------------VVALG-ANI-ICNRIPG-L : 42
NvWnt5 : M-------------------------------------------VLTSFFH---AGH-SVWSVNIPH----------Q---AYIIS-VQPGLCMNLGG-L : 38
bfWnt5 : M-----------------------------AVQVSLRVLRVLVTLLSCYTH-LGRVRATWWQMAVDSRL--YSLSRAE---LYIIG-AQP-LCTTLAG-L : 62
mmWnt5a : M-----------------------------SSKF----FLMALATFFSFAQ-VVIEANSWWSLGMNNP-----VQMSE---VYIIG-AQP-LCSQLAG-L : 55
mmWnt5b : M-----------------------------PSLL----LVVVAALLSSWAQ-LLTDANSWWSLALNP------VQRPE---MFIIG-AQP-VCSQLPG-L : 54
Nvwnt-A : M-----------------------------AVSVAVI--------TLAILL-SEQADGRRKFRDN----PVIPSARPSL--VTSEE-VTQ-FCSLMPW-M : 53
pdWnta : ---------------------------------------------------------------------------------------------------- : -
pvWnta : ---------------------------------------------------------------------------------------------------- : -
NvWnt4 : M-----------------------------IA---L----KIVILTQLVMA-SLVYSSQWLSLALTGSN------------PNRFL-SKK-NCDEIGQ-L : 48
bfWnt4 : M-----------------------------PTLNVI----LVFLLYALVST-CSATANQWLRVA--AS-------------AVSVR-TED-ACEKLHG-L : 48
mmWnt4 : M-----------------------------SPRSCL----RS-LRLLVFAV-FSAAASNWLYLAKLSS-------------VGSIS-EEE-TCEKLKG-L : 49
NvWnt1 : M------------------------------QRFS---AAILLVFMVSVC-ISNHEVQGWWNLGFGFED--L---------KNDYNIQLS-PPNQIRA-L : 53
NvWnt6 : M--------------------------------------------------------------------------------------------------- : 1
bfWnt1 : M--------------------------KLVCTFWA---VVLLFLAVVPVE-RVHAVIGRWWGIASTVAV--QEHANMVPGVARKPGSTIM-LDPKKHP-L : 66
mmWnt1 : M------------------------GLWALLPSWVST-TLLLALTALPAA-LAANSSGRWWGIVNIASS--TNLL------TDSKSLQLV-LEPSLQL-L : 64
bfWnt6 : ---------------------------------------------------------------------------------------------------- : -
mmWnt6 : M-----------------------------LPPVPSR-LGLLLLLLCP-----AHVDGLWWAVGSPLVM--D---------------PTS-ICRKARR-L : 46
 ●

* 120 * 140 * 160 * 180 * 200
HvWnt : HH-YQRTVCLNYT-DLMLSVAEGIRLGIDECQVQFKHRKWNCTINEHG---------TSVFGP--II--------------------------TTASRES : 114
bfWnt3 : VP-RQIRYCRKFH-EIMPFVADGTKLGIRECQHQFRGRRWNCTTVQGQ---------VSIFGP--VLDRGNISTTPKTLPRPRAGDSILGPAVNRASREA : 151
mmWnt3 : VP-KQLRFCRNYI-EIMPSVAEGVKLGIQECQHQFRGRRWNCTTIDDS---------LAIFGP--VL--------------------------DKATRES : 112
mmWnt3a : VP-KQLRFCRNYV-EIMPSVAEGVKAGIQECQHQFRGRRWNCTTVSNS---------LAIFGP--VL--------------------------DKATRES : 109
NvWnt3 : NS-RQQEVCRKNP-DLMEHVAHGAKYGVHECRHQFRNRRWNCSTIRES---------GSLFES--VL--------------------------SKGCREA : 109
NvWnt10 : SK-EQLKMCRRLP-DVVASALQGMQYAIHECLAQFRYRRWNCSSLEMK-------NRNPLANP--LLS--------------------------RGFRET : 112
bfWnt10 : ---------------------------------------------------------------------------------------------------- : -
mmWnt10a : SR-RQMEVCVRHP-DVAASAIQGIQIAIHECQHQFRDQRWNCSSLETR-------NKVPYESP--IFS--------------------------RGFRES : 130
mmWnt10b : SK-RQLGLCLRSP-DVTASALQGLHIAVHECQHQLRDQRWNCSALEGG-------GRLPHHSA--ILK--------------------------RGFRES : 117
NvWnt11 : SG-KQYKICRRNL-PAMLYVTAAVEMTREECQHQFQNKRWNCSTIVK----------APQFLP--DLK--------------------------RGTPEA : 106
bfWnt11 : VP-EQTQLCRRTL-EVMPAVEYAAESARKTCQEQFGNRRWNCSSIKK----------APHFMN--DLE--------------------------KGTKEA : 109
mmWnt11 : VS-AQVQLCRSNL-ELMRTIVHAARGAMKACRRAFADMRWNCSSIEL----------APNYLL--DLE--------------------------RGTRES : 111
mmWnt9a : ER-KQRRMCRRDP-GVAETLVEAVSMSALECQYQFRFERWNCTLEGR----------YR--AS--LLK--------------------------RGFKET : 122
mmWnt9b : SR-RQKQLCRREP-GLAETLRDAAHLGLLECQFQFRQERWNCSLEGR----------T----G--LLQ--------------------------RGFKET : 118
NvWnt8 : ----------------------------------------DCFLAF------------------------------------------------TANRET : 13
NvWnt8b : VL-RGR---EAYD-KLVSSIQKGAKLGLQECRSQFRNEKWNCTMAVKTKNKSTSKQNPAYVMS--MVP--------------------------HATREM : 82
bfWnt8a : IT-GPKSSFQASL-TYASSVAAGAQTAMEECKHQFSWDRWNCTDNA-----------LSMFKP--NTL--------------------------PANREA : 91
mmWnt8a : IT-RPK----AYL-TYTASVALGAQIGIEECKFQFAWERWNCPEHAF----------QFSTHN--RLR--------------------------AATRET : 85
mmWnt8b : MT-GPK----AYL-VYSSSVAAGAQSGIEECKYQFAWDRWNCPERAL----------QLSSHG--GLR--------------------------SANRET : 84
NvWnt7a : SA-KQMRFCEDKP-GTMVSISQGYDLGVEECKYQFRNKRWNCSLLGE----------ERPFGQ--RAV--------------------------PGTKEA : 95
NvWnt7b : SA-KQMRFCEDKP-GTMVSISQGYDLGVEECKYQFRNKRWNCSLLGE----------ERPFGQ--RAV--------------------------PGTKEA : 109
AmqWnt : NS-SQQTFCNYNR-KIVNSIAIGTRRGIVACQQNFANWRWNCTTFTG----------ENLFGA--FVK--------------------------NNTRET : 109
NvWnt16 : SL-QQLRMCLQKP-DVIPSVSQGANIGIHECKKQFKYERWNCSTS----------NDPTVFGT--LLK--------------------------IAHKES : 114
atWnt16 : VM-QQQKVCQAHP-AVIKAVSSGAKRGIHECQNQFRHDRWNCTIE----------GGESVFDH--TLQ--------------------------RGSRET : 113
mmWnt16 : NS-RQKELCKRKP-YLLPSIREGARLGIQECRSQFRHERWNCMVATTTSTQ---LATAPLFGY--ELS--------------------------SGTKET : 119
NvWnt2 : IS-IQRQMCLDNP-DVMVSIGKGAKLGVEECQHQFRDQRWNCSTVNG---------DATVFGK--VMR--------------------------RASRET : 109
mmWnt2 : VS-RQRQLCHRHP-DVMRAIGLGVAEWTAECQHQFRQHRWNCNTLDR---------DHSLFGR--VLL--------------------------RSSRES : 108
mmWnt2b : VS-RQRQLCQRYP-DIMRSVGEGAREWIRECQHQFRHHRWNCTTLDR---------DHTVFGR--AML--------------------------RSSREA : 137
bfWnt7a : ---------------------------------------------------------------------------------------------------- : -
mmWnt7a : AP-RQRAICQSRP-DAIIVIGEGSQMGLDECQFQFRNGRWNCSALGE----------RTVFGK--ELK--------------------------VGSREA : 104
mmWnt7b : AP-RQRAICQSRP-DAIIVIGEGAQMGIDECQHQFRFGRWNCSALGE----------KTVFGQ--ELR--------------------------VGSREA : 104
bfWnt7b : VP-RQRAICQTRP-DLIVAIGEGAQRGIDECRYQFRHSRWNCTGMDN----------DNVFGR--ELR--------------------------IGSKEA : 102
NvWnt5 : TR-EQIDLCQKNI-DHMASVGLGAKMAIQECQFQYQYEKWNCSIPDAE--------KSSLFER--ITSK------------------------DVATREA : 102
bfWnt5 : SS-GQRKLCNLYQ-DHMSSVGIGARQGIEECQHQFRDRRWNCTTSD----------EDSVFGR--IVN--------------------------IGSREA : 122
mmWnt5a : SQ-GQKKLCHLYQ-DHMQYIGEGAKTGIKECQYQFRHRRWNCSTVD----------NTSVFGR--VMQ--------------------------IGSRET : 115
mmWnt5b : SP-GQRKLCQLYQ-EHMSYIGEGAKTGIRECQHQFRQRRWNCSTVD----------NTSVFGR--VMQ--------------------------IGSRET : 114
Nvwnt-A : KP-HQKQKCLLEP-LVMPSVQRGVGMALEECPSHYSDHKWNCSGVN----------TAQVFQERGILK--------------------------TNTKES : 115
pdWnta : -------------------------------EYQFSDRRWNCTTFN----------NTSVFGK--VLS--------------------------KKTRER : 31
pvWnta : ---------------------------------------------------------------------------------------------------- : -
NvWnt4 : SNHRQVQVCKRNI-QVMDSVKDGASVALFECQHQFRYRPWNCTTVQFS--------RSPVFGN--SIN--------------------------GGTREA : 111
bfWnt4 : IS-RQVQICKRNV-EVMDSVKEGARMSIEECQFQFRHRRWNCSTLINR--------RGPVFGK--VLE--------------------------EGTREA : 110
mmWnt4 : IQ-RQVQMCKRNL-EVMDSVRRGAQLAIEECQYQFRNRRWNCSTLDS----------LPVFGK--VVT--------------------------QGTREA : 109
NvWnt1 : TQ-KQIRISRRYP-ELIQYIAGGARTAIHECQHQFRNRKWNCSAHSPE----------NVFGK--IL--------------------------KRACRET : 113
NvWnt6 : ---------------------------------------------------------------------------------------------------- : -
bfWnt1 : NK-KQRRLVRRNPGDAGEHRDRRPMLAIKECHHQFSKWRWNCPVNTSD-------HVNSVFGN--IL--------------------------LRGCTQT : 130
mmWnt1 : SR-KQRRLIRQNP-GILHSVSGGLQSAVRECKWQFRNRRWNCPTAPGP----------HLFGK--IV--------------------------NRGCRET : 124
bfWnt6 : ----------------------------RGCDYQMK---------------------------------------------------------------- : 8
mmWnt6 : AG-RQAELCQAEP-EVVAELARGARLGVRECQFQFRFRRWNCSSH------------SKAFGR--VL--------------------------QQDIRET : 104
 ● ● ●

* 220 * 240 * 260 * 280 * 300
HvWnt : AFISGIISAGVAFSVTESCAEGKS-VHCRCDNSVR-------------GQT---------------------------------------DEGWRWGGCN : 161
bfWnt3 : AFVHAITSAGVAYSVTKACAEGTS-PDCGCDNRHK-------------GPP---------------------------------------GEGWRWGGCS : 198
mmWnt3 : AFVHAIASAGVAFAVTRSCAEGTS-TICGCDSHHK-------------GPP---------------------------------------GEGWKWGGCS : 159
mmWnt3a : AFVHAIASAGVAFAVTRSCAEGSA-AICGCSSRLQ-------------GSP---------------------------------------GEGWKWGGCS : 156
NvWnt3 : AFVHAVTAAGVAHSVTDACSKGRI-ESCDCDRNLS-------------GRS---------------------------------------SKGWTWSGCN : 156
NvWnt10 : AFVHAILSAGMTSSVARACSMGKL-AKCGCDESLR-------------GRG----------------------------------------TGWEWGGCG : 158
bfWnt10 : ---------------------------------------------------------------------------------------------------- : -
mmWnt10a : AFAYAIAAAGVVHAVSNACALGKL-KACGCDASRR-------------GDEEAFRRKLHRLQLDALQRGKGLSHGVPEH-PAILPASPGLQDSWEWGGCS : 215
mmWnt10b : AFSFSMLAAGVMHAVATACSLGKL-VSCGCGWKGS-------------GEQDRLRAKL--LQLQALSRGKTFPISQPSPVPGSV-PSPGPQDTWEWGGCN : 200
NvWnt11 : AFVYALSAAALTYSITQACGMKRL-KPCKCGTNPK-------------FK---------------------------------------HPDG-EWGGCH : 152
bfWnt11 : AYVHALSSAAVVHTVARACAAGYL-KACTCARNPG-------------EK---------------------------------------PDGNYTWGGCG : 156
mmWnt11 : AFVYALSAATISHTIARACTSGDL-PGCSCGPVPG-------------EP---------------------------------------PGPGNRWGGCA : 158
mmWnt9a : AFLYAISSAGLTHALAKACSAGRM-ERCTCDEAPD-------------LE---------------------------------------NREAWQWGGCG : 169
mmWnt9b : AFLYAVSAAALTHALARACSAGRM-ERCTCDDSPG-------------LE---------------------------------------SRQAWQWGVCG : 165
NvWnt8 : AFVHSINTAAVTYFLTRDCRRGIF-RNCACVRQT--------------------------------------------------------GQAGEWRGCN : 56
NvWnt8b : AFAHGISAAGVTFALTMDCRLGAF-EDCSCIHGKS-------------E----------------------------------------GNKGNWWGGCN : 128
bfWnt8a : SFVHAISAAGVMYVLTRNCSKGAF-EQCGCDLTNN-------------GKK--------------------------------------AEGGWTWGGCS : 139
mmWnt8a : SFIHAIRSAAIMYAVTKNCSMGDL-ENCGCDESQN-------------GKT--------------------------------------GGHGWIWGGCS : 133
mmWnt8b : AFVHAISSAGVMYTLTRNCSLGDF-DNCGCDDSRN-------------GQL--------------------------------------GGQGWLWGGCS : 132
NvWnt7a : AFTHAIISAGIVQAVTLACTQNPT--GCGCDRNKD-------------GI---------------------------------------SREGWKWGGCS : 141
NvWnt7b : AFTHAIISAGIVQAVTLACTQNPT--GCGCDRNKD-------------GI---------------------------------------SREGWKWGGCS : 155
AmqWnt : AVINALLTAGAERQIALDCRDEKL-PNCTCQINGDN------------GV---------------------------------------VNSTFFLYECS : 157
NvWnt16 : AFVYAITSAGVVHAVGKSCSKGNL-TECSCESKRG-------------AR-N-------------------------------------QPKGWEWGGCS : 162
atWnt16 : AFIYAITSAGATHAVTQACSAGNL-TDCSCDTSRQ-------------GQ-S-------------------------------------MPEGWKWGGCS : 161
mmWnt16 : AFIYAIMAAGLVHSVTRSCSAGNM-TECSCDTTLQ-------------NGGS-------------------------------------PSEGWHWGGCS : 168
NvWnt2 : AFVYAISSAGVVHEVTRSCSLGEL-KDCSCRN-KK-------------GRSR---------------------------------------KGFEWGGCS : 155
mmWnt2 : AFVYAISSAGVVFAITRACSQGEL-KSCSCDPKKK-------------GSAK------------------------------------DSKGTFDWGGCS : 158
mmWnt2b : AFVYAISSAGVVHAITRACSQGEL-SVCSCDPYTR-------------GRHH------------------------------------DQRGDFDWGGCS : 187
bfWnt7a : ---------------------------------------------------------------------------------------------------- : -
mmWnt7a : AFTYAIIAAGVAHAITAACTQGNL-SDCGCDKEKQ-------------GQYH------------------------------------R-DEGWKWGGCS : 153
mmWnt7b : AFTYAITAAGVAHAVTAACSQGNL-SNCGCDREKQ-------------GYYN------------------------------------Q-AEGWKWGGCS : 153
bfWnt7b : AFTYAISSAALVHAIVTACSQGNI-SDCGCDRTKE-------------GDL-------------------------------------N-DEGWKWGGCS : 150
NvWnt5 : ALTYAISSAGVVWALARACTEGNL-STCSCSRERR-------------PLD--------------------------------------LNKEYQWGGCG : 150
bfWnt5 : SFTYAIAAAGVVNAVSRACREGEL-TTCGCSRAKR-------------PKD--------------------------------------LNRDWLWGGCG : 170
mmWnt5a : AFTYAVSAAGVVNAMSRACREGEL-STCGCSRAAR-------------PKD--------------------------------------LPRDWLWGGCG : 163
mmWnt5b : AFTYAVSAAGVVNAISRACREGEL-STCGCSRAAR-------------PKD--------------------------------------LPRDWLWGGCG : 162
Nvwnt-A : AFVFALTSAGVSFQITKGCSLGNW-EQCGCDTQVR-------------GRVQT-----------------------------------KDEASWEWGGCS : 166
pdWnta : AYIYAVSSAGVMYSITKACAKGDL-HMCSCDTSIR-------------NKET--------------------------------------KGEFLWGGCS : 79
pvWnta : ---------------------------------------------------------------------------------------------------- : -
NvWnt4 : AFVHAISSAGVAYAVTQACSSGRLGQKCGCDRKTR-------------GQA----------------------------------------DGFNWGGCS : 158
bfWnt4 : AFVHSISAAGVAHAVTRACSSGEL-ERCGCNRTVR-------------GTI---------------------------------------PEGFHWAGCS : 157
mmWnt4 : AFVYAISSAGVAFAVTRACSSGEL-EKCGCDRTVH-------------GVS---------------------------------------PQGFQWSGCS : 156
NvWnt1 : AFTYAITAAGVSHAIARACGEGKL-SACSCDQRYR-------------GVS---------------------------------------KQGWQWGGCS : 160
NvWnt6 : --------------------------------GLD-------------EAE---------------------------------------RHDFGRRGCQ : 17
bfWnt1 : AFIYAVMSAAVAHEVGRNCAEGTI-ETCSCDYRSK-------------GPA---------------------------------------GEDWEWGGCS : 177
mmWnt1 : AFIFAITSAGVTHSVARSCSEGSI-ESCTCDYRRR-------------GPG---------------------------------------GPDWHWGGCS : 171
bfWnt6 : ------------------------------------------------GES--------------------------------------PDGSWEWGGCG : 22
mmWnt6 : AFVFAITAAGASHAVTQACSMGEL-LQCGCQAPRGRAPPRPSGLLGTPGPP------------------------------GPT-GSPDASAAWEWGGCG : 172
 ● ● ● ●

* 320 * 340 * 360 * 380 * 400
HvWnt : R-PITYGIWFSQLFIDQVEKIV----KKRKDPRK--IMNLHNNKAGREV---IKNLLQTECKCHGT-SGNCNLKTCWRSQPHFSEIGKILKEKYDSAHEM : 250
bfWnt3 : E-DVLFGTKFSRDFVDARIR-------GRRDGRS--AMDRHNNEAGRQS---IMKNLQLKCKCHGL-SGSCEIKTCWWAQPDFRTVGNVLKDKYDSASEM : 284
mmWnt3 : E-DADFGVLVSREFADARE--------NRPDARS--AMNKHNNEAGRTT---ILDHMHLKCKCHGL-SGSCEVKTCWWAQPDFRAIGDFLKDKYDSASEM : 244
mmWnt3a : E-DIEFGGMVSREFADARE--------NRPDARS--AMNRHNNEAGRQA---IASHMHLKCKCHGL-SGSCEVKTCWWSQPDFRTIGDFLKDKYDSASEM : 241
NvWnt3 : S-NIKFGVWFSKQFTEARER--------GDDLRQ--IMNRHNSRAGRKA---LEELVWRKYKCHGL-SGSCSMKTCWMQQANFRQIGDHLKVKYDSAVEM : 241
NvWnt10 : D-NIDYGIETSAKFLDSREKG--------RDLHS--MMNMHNNMVGRTT---LSENAKTKCKCHGM-CGSCSVKTCWKTVPDIREIGDRLMEKYDHATTI : 243
bfWnt10 : -----------------------------------------------------------------M-SGSCNLKTCWKATPDFREVGVILKERFDYATQI : 34
mmWnt10a : P-DVGFGERFSKDFLDSREPH--------RDIHA--RMRLHNNRVGRQA---VMENMRRKCKCHGT-SGSCQLKTCWQVTPEFRTVGALLRNRFHRATLI : 300
mmWnt10b : H-DMDFGEKFSRDFLDSREAP--------RDIQA--RMRIHNNRVGRQV---VTENLKRKCKCHGT-SGSCQFKTCWRAAPEFRAIGAALRERLSRAIFI : 285
NvWnt11 : D-NIARGMRFSKDFTDAVEAQRMR--KHKSMAVA--LMNLHNNGVGRKA---VHSRLEFHCRCHGV-SGGCTAKTCIRRLGDFRLVADLLKNRYARIVYV : 243
bfWnt11 : D-NVKFGLEFGSRFADAPMR---K--KKRSHTQT--LMNLHNSEAGRLA---VQNTMTTKCKCHGV-SGSCNVKTCWKSLADLTEISHELAEKYSYAIKV : 244
mmWnt11 : D-NLSYGLLMGAKFSDAPMKV--K--KTGSQANK--LMRLHNSEVGRQA---LRASLETKCKCHGV-SGSCSIRTCWKGLQELQDVAADLKTRYLSATKV : 247
mmWnt9a : D-NLKYSSKFVKEFLG-R--------RSSKDLRA--RVDFHNNLVGVKASWVIKAGVETTCKCHGV-SGSCTVRTCWRQLAPFHEVGKHLKHKYETSLKV : 256
mmWnt9b : D-NLKYSTKFLSNFLGPK--------RGSKDLRA--RADAHNTHVGIKA---VKSGLRTTCKCHGV-SGSCAVRTCWKQLSPFRETGQVLKLRYDTAVKV : 250
NvWnt8 : D-NVKFGEVLSKHFLNARHVD-------KRKARA--VIHLHNNAVGRKA---VKKTLKQQCKCHGV-SGGCSSKSCWKTLPLFSEIGDYLKAKYQQAQKV : 142
NvWnt8b : E-NVKFGEVMARHFLEALQSG--------KDERS--LLNVHNNEVGRKA---VRATLKRECRCHGI-SGSCSTRTCWRKLSSFAEVGQYLVEKYSTAKRV : 213
bfWnt8a : D-DIAFGERISKMYSDGVENG--------QDARA--AMNLHNNDVGRKA---VRQTMKRVCKCHGV-SGSCTTKTCWLQLADFRAIGVFLKKKYKKADKV : 224
mmWnt8a : D-NVEFGEKISRLFVDSLEKG--------KDARA--LVNLHNNRAGRLV---LRASTKRTCKCHGI-SGSCSIQTCWLQLADFRQMGNYLKAKYDRALKI : 218
mmWnt8b : D-NVGFGEAISKQFVDALETG--------QDARA--AMNLHNNEAGRKA---VKGTMKRTCKCHGV-SGSCTTQTCWLQLPEFREVGAHLKEKYHAALKV : 217
NvWnt7a : V-NIGHGLAVAKEFLNANDAV--------RSDIA--LMNRHNNEVGREV---VNRSLLTECTCHGP-SASCVTRTCSQALPSPRAVSNRLKALYDTARRA : 226
NvWnt7b : V-NIGHGLAVAKEFLNANDAV--------RSDIA--LMNRHNNEVGREV---IRNNLDLSCKCHGP-SGSCNTKTCWKSVPSFRMVGEKLRALYESRQA- : 239
AmqWnt : F-DIAKAHDIMSKFLETPS----------KDDTA--IIAEHNHNVGSNL---VGQRYR-KCRCTGF-SGSCSVQTCYFASPDIDTIGQRVREKYGSSVEV : 239
NvWnt16 : D-NVNYGVWLSKTFVDAPEKADRRAR-SQRKARA--MMNLHNNEAGREA---VLALMRVQCRCHGV-SSSCAVKTCSKSLPKFEEVGEALKAEYKDAIRA : 254
atWnt16 : D-NVRYGMMFARQFVDAPERAE-----RKRDVRA--LMNLHNNNAGRLA---IARQMELKCRCHGV-SGSCELKTCWNKLPSFEQVGHFLKSKYDNSIQV : 249
mmWnt16 : D-DVQYGMWFSRKFLDLPIRNT-----TGKESRVLLAMNLHNNEAGRQA---VAKLMSVDCRCHGV-SGSCAVKTCWKTMSSFEKIGHFLKDKYENSIQI : 258
NvWnt2 : D-NIQYGLNFAKAFVDSREVE--------KDARA--LMNLHNNHVGRRV---VKTNMSLDCKCHGV-SGSCSVRTCWKSISSFRIVGQHLREKYTTAVQV : 240
mmWnt2 : D-NIDYGIKFARAFVDAKERK-------GKDARA--LMNLHNNRAGRKA---VKRFLKQECKCHGV-SGSCTLRTCWLAMADFRKTGDYLWRKYNGAIQV : 244
mmWnt2b : D-NIHYGVRFAKAFVDAKEKR-------LKDARA--LMNLHNNRCGRTA---VRRFLKLECKCHGV-SGSCTLRTCWRALSDFRRTGDYLRRRYDGAVQV : 273
bfWnt7a : ----------------------------------------------------------QECKCHGV-SGSCTTKTCWTTLPKFRELGYILKDKYHEAVQV : 41
mmWnt7a : A-DIRYGIGFAKVFVDAREIK--------QNART--LMNLHNNEAGRKI---LEENMKLECKCHGV-SGSCTTKTCWTTLPQFRELGYVLKDKYNEAVHV : 238
mmWnt7b : A-DVRYGIDFSRRFVDAREIK--------KNARR--LMNLHNNEAGRKV---LEDRMKLECKCHGV-SGSCTTKTCWTTLPKFREVGHLLKEKYNAAVQV : 238
bfWnt7b : A-DVKYGLRFCKKFVDAREVE--------QNARA--LMNLHNNEAGRKV---IDQHTRLECKCHGV-SGSCTMKTCWITLPRFREVGNILKEKYHHDAQL : 235
NvWnt5 : D-NIEYAVKFGREFMEAGEDHRPTEEDRKKYART--LMNLHNNNLGRRV---VKDISVVECKCHGV-CGSCNLKTCWRQLVEFREIGNALHDKYDAAVQV : 243
bfWnt5 : D-DVEYGYYFAREFVDAQEKQIIPTPGSQAHARQ--LMNMHNNEAGRKL---TFSNARVACKCHGV-SGSCSLKTCWQQLADFRTVGNLLKDKYDGANEV : 263
mmWnt5a : D-NIDYGYRFAKEFVDARERERIHAKGSYESARI--LMNLHNNEAGRRT---VYNLADVACKCHGV-SGSCSLKTCWLQLADFRKVGDALKEKYDSAAAM : 256
mmWnt5b : D-NVEYGYRFAKEFVDAREREKNFAKGSEEQGRA--LMNLQNNEAGRRA---VYKMADVACKCHGV-SGSCSLKTCWLQLAEFRKVGDRLKEKYDSAAAM : 255
Nvwnt-A : E-NVGHGDDFSRKFMDPEPPR--------KELEY--LLVKHNNEAGRKA---LKDNMGKTCKCHGV-SGSCTVKICWRTMPNFSVVPQLLRKKFDQATKV : 251
pdWnta : H-NVKFGERFTREFVDTKENG--------EDPDG--LMNIWNNGAGRKT---IKSSMRLLCKCYGVFSGSCSVKICWRTMAPFREIGRHLKQKFDGASLV : 165
pvWnta : ------RTSGSPGLQDSARVR--------LNADG--LMNVWNNGAGRKT---VKEELDLICKCHGV-SGSCSVKICWRKMKTFRAIGTTLKNRFDGASLV : 80
NvWnt4 : D-DIDFGMTFATRFVDARERGSG----IGSPARV--LMNLHNNRGGRLA---VRKFMDLQCKCHGV-SGSCNIKTCWRALPNFRIVGDYIKEKFDGATEV : 247
bfWnt4 : D-NFAFGAAFSQTFVDARERGRV----AATSSRA--LMNLHNNEAGRRN---LVDHMKTECKCHGV-SGSCELKTCWRAMPPFREVGARLKEKFDGATEV : 246
mmWnt4 : D-NIAYGVAFSQSFVDVRERSKG-----ASSSRA--LMNLHNNEAGRKA---ILTHMRVECKCHGV-SGSCEVKTCWRAVPPFRQVGHALKEKFDGATEV : 244
NvWnt1 : D-NIHFADNFSKRFVDAQEK-------GR-DFRA--QINLHNNEAGRAA---VRNNMMLECKCHGL-SEACTVKTCWKRLPDFRLVGDDLKAKFDDASMV : 245
NvWnt6 : HVYTQFGFDTAKQFMDPMG--------SR-DAKA--LISRHNNKAGRLA---VKNHMEKKCRCHGL-SQTCQMKTCWWELPAFRSVSDRIKTHFDGAVKV : 102
bfWnt1 : D-NVEFGKQFAKQFVDAGEK-------TKDSVRY--LVNMHNNEAGRVA---VAENLRRECKCHGM-SGSCTLKTCWMRLPNFRDVGDSLKEKFDGASKV : 263
mmWnt1 : D-NIDFGRLFGREFVDSGEK-------GR-DLRF--LMNLHNNEAGRTT---VFSEMRQECKCHGM-SGSCTVRTCWMRLPTLRAVGDVLRDRFDGASRV : 256
bfWnt6 : D-DIDFGYTKSREFMDAQTR-------HRSDIRT--LLTLHNNEAGRLA---EKNFMRTECKCHGL-SGSCAVKTCWKKMPIFREVGVRLKERFNGAFQV : 108
mmWnt6 : D-DVDFGDEKSRLFMDAQHKR------GRGDIRA--LVQLHNNEAGRLA---VRSHTRTECKCHGL-SGSCALRTCWQKLPPFREVGARLLERFHGASRV : 259

* 420 * 440 * 460 * 480 * 500
HvWnt : EF-LYKVKAN-G----ERKIKDLI------PKYKEYLPPSSLDFIYYEESPNYCVKNETLGIAGTKGRSCNITS-S-----GVDG-CELMC--CQRGYNV : 329
bfWnt3 : AV-ERHRKPS-G------MVDSLY------PRYSFFKAPGKDDLIYFEVSPNFCEPNNSTGSLGTKGRECNITS-Q-----GIDG-CQLMC--CGRGWNT : 361
mmWnt3 : VV-EKHRESR-G------WVETLR------AKYALFKPPTERDLVYYENSPNFCEPNPETGSFGTRDRTCNVTS-H-----GIDG-CDLLC--CGRGHNT : 321
mmWnt3a : VV-EKHRESR-G------WVETLR------PRYTYFKVPTERDLVYYEASPNFCEPNPETGSFGTRDRTCNVSS-H-----GIDG-CDLLC--CGRGHNA : 318
NvWnt3 : TT-KVNRR---G----KK---RLK------PKYSHFKKPSDKDLIYFETSPNYCDKNVTVGSLGTSGRQCNYTS-N-----GIDG-CELLC--CGRGHNI : 315
NvWnt10 : GM-GNGRLR-------LHLTRRKA-----------RRSSVGRALVYYEDSPNYCIENKELGIFGTRGRICSPES-L-----DTDN-CQNLC--CERGYTT : 315
bfWnt10 : KV-SNDNYG-------RLETIYAN------------RPPFATDLVYFDRSPDFCDRNRELETPGTRGRICNKTS-T-----GPDS-CAALC--CGRGFNI : 105
mmWnt10a : RP-HNRNGG-------QLEPGPAGAPSPAPGTPGLRRRASHSDLVYFEKSPDFCEREPRLDSAGTVGRLCNKSS-T-----GPDG-CGSMC--CGRGHNI : 383
mmWnt10b : DT-HNRNSG-------AFQPR-------------LRPRRLSGELVYFEKSPDFCERDPTLGSPGTRGRACNKTS-R-----LLDG-CGSLC--CGRGHNV : 355
NvWnt11 : ES-KTKSKRKA------R--VLKS--------KRGRRRYTSSDLVALQGSPNYCHKNRKRGTAGTHGRLCDPTK-RR----GEGS-CAYLC--CGRGHRT : 318
bfWnt11 : VK-RKIGTRQQ---------LVPE--------DRRSRRHGSGDLIFVENSPNYCMVNNRKGSYGTTGRLCNKTS-V-----GPDS-CQTMC--CGRGYND : 317
mmWnt11 : VH-RPMGTRKH---------LVPK--------DLDIRPVKDSELVYLQSSPDFCMKNEKVGSHGTQDRQCNKTS-N-----GSDS-CDLMC--CGRGYNP : 320
mmWnt9a : GS-TTNEATGE------AGAISPPR-GRASGSGGGDPLPRTPELVHLDDSPSFCLAGRF--SPGTAGRRCHREK----------N-CESIC--CGRGHNT : 333
mmWnt9b : SS-ATNEALGR------LELWAPAK-PGGPA---KGLAPRPGDLVYMEDSPSFCRPSKY--SPGTAGRVCSRDS----------S-CSSLC--CGRGYDT : 324
NvWnt8 : RL-HTNKLVLK---------LPSRV-------FAPLTKKARRSLVFLKPSPDYCHRDTKKGSTGVLGRECSSDS-P-----NYLE-CIQMCTSCDYRVEK : 218
NvWnt8b : IF-QNGNF-YE---------LTMLG--------TRPISKKDNNFIYSESSPDYCQRNMTVGSAGVLGRECEGSK-D-----ELVR-CRQLCDSCRFDTQE : 287
bfWnt8a : DY-VRGQLTE----------NNSAS--------SKRNTGLKKDMVFLEDSPDYCAMPLTVGSRGTLGRECLRGGGKNMDKYEKKS-CKRLCKDCGYVPKR : 304
mmWnt8a : EM-DKRQLRAGN----RAEGRWALT--------EAFLPSTEAELIFLEGSPDYCNRNASLSIQGTEGRECLQNA-RSASRREQRS-CGRLCTECGLQVEE : 303
mmWnt8b : DL-LQGA---GN----SAAGRGAIA--------DTFRSISTRELVHLEDSPDYCLENKTLGLLGTEGRECLRRG-RALGRWERRS-CRRLCGDCGLAVEE : 299
NvWnt7a : TV-YLSSLVR---------------------PDDKVEKVKPMDLAYLKDSPNYCTKNTLSKLPGTLGGNCKISD-D---SQEEEN-CDVMC--CGRGYDT : 297
NvWnt7b : TV-KVVAAMK-T----SKGGQVPAY-IVVKGTNKVVKPNSSANLVYLDNSPSYCNKIKSLKVPGTVGRVCSRTP-E---SAEDVS-CEVMC--CGRGYSV : 325
AmqWnt : TV-NAS-----------NSALQPVV-QTIN--------NHDNELVYLKRSPTFCNQDTTYGILGTVGRQCSNN------LGDPDS-CDIIC--CGRGHIT : 309
NvWnt16 : VY-IKRKR-K-------LKR-----------KDNKKLRIPSSSLVYLDESPNYCYRDKKLGIDGTSGRECNKNS-S-----GVDG-CDLLC--CGSGYNT : 325
atWnt16 : SL-KAKRRLR-------RRG-----------KVKRKVPVQKEDLVHIHRSPNYCIEDFKRGILGTSGRRCNRTA-K-----GPQS-CNLLC--CGRGYNT : 321
mmWnt16 : SD-KTKRKMR-------RRE-----------KDQRQTPILKDDLLYVHKSPNYCVENKKLGIPGTQGRECNRTS-G-----GADG-CNLLC--CGRGYNT : 330
NvWnt2 : TV-GQSGGEL-------TN------------AEVSYKKPSRDDLVYLEDSPNYCMVDSNTGSLGTSGRECNGSA-S-----DTTGACSLLC--CGRGFNT : 312
mmWnt2 : VM-NQDGTGF-------TV------------ANKRFKKPTKNDLVYFENSPDYCIRDREAGSLGTAGRVCNLTS-R-----GMDS-CEVMC--CGRGYDT : 315
mmWnt2b : TA-TQDGANF-------TA------------ARQGYRHATRTDLVYFDNSPDYCVLDKAAGSLGTAGRVCSKTS-K-----GTDG-CEIMC--CGRGYDT : 344
bfWnt7a : EP-VRASRNK-------RQTFLKI------KKPLSYRKPMDTDLVYIEKSPNYCEEDPVTGSVGTQGRMCNKTA-Q-----QSDG-CDLMC--CGRGYNT : 118
mmWnt7a : EP-VRASRNK-------RPTFLKI------KKPLSYRKPMDTDLVYIEKSPNYCEEDPVTGSVGTQGRACNKTA-P-----QASG-CDLMC--CGRGYNT : 315
mmWnt7b : EV-VRASRLR-------QPTFLRI------KQLRSYQKPMETDLVYIEKSPNYCEEDAATGSVGTQGRLCNRTS-P-----GADG-CDTMC--CGRGYNT : 315
bfWnt7b : VEAVRARRTR-------RPTFLKL------KNSRTFEKPREISLVYLRGSPNYCERDEATGSLGTHGRRCNRTS-P-----YQDG-CDLMC--CGRGYNT : 313
NvWnt5 : AL-KRKEGRSL-----LLPMRSRHY----SQRKAKSAQETRDELVYIDKSPDFCSKNAAHGAQGTRGRKCIKES-L-----GKDG-CNLLC--CSRGYKM : 324
bfWnt5 : KL-IKRGKRYR-----LDRRN------------PRFNVFTDEDLVYLNKSPDYCNADPTIGSLGTHGRECNKTG-L-----GTDG-CNLMC--CGRGYNT : 336
mmWnt5a : RL-NSRGK--------LVQVN------------SRFNSPTTQDLVYIDPSPDYCVRNESTGSLGTQGRLCNKTS-E-----GMDG-CELMC--CGRGYDQ : 326
mmWnt5b : RI-TRQGK--------LELAN------------SRFNQPTPEDLVYVDPSPDYCLRNETTGSLGTQGRLCNKTS-E-----GMDG-CELMC--CGRGYDR : 325
Nvwnt-A : KA-NDKKTK-------LQRITRG---KRGKKKRQKGRRPSAGDLVFAEKSPKFCIPNPELGILGTRGRVCDANA-K-----DNRG-CKKMC--CNRGYDT : 331
pdWnta : TI-NSKKSK-------LK------------PVDRRIKKPAKDELVYMEDSPDYCEYDPGIGSLGTRGRQCNKTS-Y-----GLDG-CSLMC--CGRGYYT : 236
pvWnta : KM-DKRKKR-------LK------------RLSRLQKRPTKKDLVYLQESPDFCEHNLEFGSLGTRGRQCNKTS-Y-----GLDG-CRLMC--CGRGHR- : 150
NvWnt4 : EY-KLIGGKHV-----LV------------PKNRKYKPHTQMDLVYLVQSPDFCEPNPKTGSLGTQGRICNRTS-Q-----AIDG-CDLMC--CGRGYVS : 320
bfWnt4 : QQ-KKIGSRRE-----LV------------PLNSDFKPHSSSDLVYLDASPDFCVRDTKVGSMGTVGRVCNKTS-K-----AIDG-CELLC--CGRGYNT : 319
mmWnt4 : EP-RRVGSSRA-----LV------------PRNAQFKPHTDEDLVYLEPSPDFCEQDIRSGVLGTRGRTCNKTS-K-----AIDG-CELLC--CGRGFHT : 317
NvWnt1 : EY-QQNNNNRNSNRNRNEDPALFI------PSKPYLRRPTVYDLGYYEHSPNFCERNPSAGSLGTQGRECNTTS-M-----GTDG-CELMC--CGRGFTT : 329
NvWnt6 : YV-DNKGER-------------II------AEESTVKPPTEEDLVYTTRSPDFCNSEYRTGSLGTRGRTCNETS-Q-----GTGG-CELLC--CGRGYER : 173
bfWnt1 : AF-PDIGNNRGS----RAKVTGLV------PKNSRHKFPTDNDLVYHERSPNFCRNNPRLGFEGTRGRECNVTS-R-----GLDG-CDLLC--CGRGYAT : 343
mmWnt1 : LY-GNRGSNRAS----RAELLRLE------PEDPAHKPPSPHDLVYFEKSPNFCTYSGRLGTAGTAGRACNSSS-P-----ALDG-CELLC--CGRGHRT : 336
bfWnt6 : MG-SNNGK-------------YLI------PVGDTIKAPTAEDLVYTNESPNFCKRNRKTGSQGTKGRACNATS-M-----GIGG-CDLLC--CGRGYKE : 179
mmWnt6 : MG-TNDGK-------------ALL------PAVRTLKPPGRADLLYAADSPDFCAPNRRTGSPGTRGRACNSSA-P-----DLSG-CDLLC--CGRGHRQ : 330
 ● ● ● ● ●

* 520 * 540 * 560
HvWnt : NIVQKTHSCECK-FVWCCKVSCNSCIKM-TPEYTCK----------------------------- : 363
bfWnt3 : RTEMRTEKCHCQ-FHWCCYVTCQECQKK-HQVHTCK----------------------------- : 395
mmWnt3 : RTEKRKEKCHCV-FHWCCYVSCQECIRI-YDVHTCK----------------------------- : 355
mmWnt3a : RTERRREKCHCV-FHWCCYVSCQECTRV-YDVHTCK----------------------------- : 352
NvWnt3 : QQAKITRNCNCV-FKWCCEVKCERCKEV------------------------------------- : 342
NvWnt10 : KKLQVTKRCRCH-FSWWCYLICDTCRET-STVSICS----------------------------- : 349
bfWnt10 : FRQTRVERCNCK-F--------------------------------------------------- : 118
mmWnt10a : LRQTRSERCHCR-FHWCCFVVCEECRIT-EWVSVCK----------------------------- : 417
mmWnt10b : LRQTRVERCHCR-FHWCCYVLCDECKVT-EWVNVCK----------------------------- : 389
NvWnt11 : EEVVHEERCECK-YIWCCYVKCQTCRKR-VRESRCL----------------------------- : 352
bfWnt11 : FTVTVTERCNCK-YHWCCYVTCDQCTRT-EKKYMCK----------------------------- : 351
mmWnt11 : YTDRVVERCHCK-YHWCCYVTCRRCERT-VERYVCK----------------------------- : 354
mmWnt9a : QSRVVTRPCQCQ-VRWCCYVECRQCTQR-EEVYTCKG---------------------------- : 368
mmWnt9b : QSRMVVFSCHCQ-VQWCCYVECQQCAQQ-ELVYTCKR---------------------------- : 359
NvWnt8 : KLAVRSSKCNCK-FVWCCDIKCSECKKL-VAVTKCVR---------------------------- : 253
NvWnt8b : FTEIKNTFCNCK-FHWCCKVKCMTCKET-TRKTRCVARQQA-------------------L---- : 327
bfWnt8a : ITTEVTSSCNCK-FHWCCSVKCSQCTKT-VTKYICVQRESSKNRRKNVRKKSKQRRRNRGNN--- : 364
mmWnt8a : RRAEAVSSCDCN-FQWCCTVKCGQCRRV-VSRYYCTR------------PVGSARPRGRGKDSAW : 354
mmWnt8b : RRAETVSSCNCK-FHWCCAVRCEQCRRR-VTKYFCSRAERP--------PRGAAHKPGKNS---- : 350
NvWnt7a : HLITKRWQCRCK-FVWCCNVRCSTCSKR-DIVQTCK----------------------------- : 331
NvWnt7b : REQIKEWKCHCK-FHWCCRVECAKCSKK-LMVHTCQ----------------------------- : 359
AmqWnt : VTATQPKQC-CS-FIYCCRIECQDCGEETFTEYFCK----------------------------- : 343
NvWnt16 : QTVRSVHSCHCR-FIWC------------------------------------------------ : 341
atWnt16 : QVHRRLERCQCK-FHWCCYVKCKTCETM-EEIYTCK----------------------------- : 355
mmWnt16 : HVVRHVERCECK-FIWCCYVRCRRCESM-TDVHTCK----------------------------- : 364
NvWnt2 : IQIEEEYKCHCK-FHWCCYVKCQTCRRT-VDKHICKAPSQP------------------------ : 351
mmWnt2 : SHVTRMTKCECK-FHWCCAVRCQDCLEA-LDVHTCKAPKSADWATPT------------------ : 360
mmWnt2b : TRVTRVTQCECK-FHWCCAVRCKECRNT-VDVHTCKAPKKAEWLDQT------------------ : 389
bfWnt7a : HQYSRVWQCNCK-FHWCC----------------------------------------------- : 135
mmWnt7a : HQYARVWQCNCK-FHWCCYVKCNTCSER-TEMYTCK----------------------------- : 349
mmWnt7b : HQYTKVWQCNCK-FHWCCFVKCNTCSER-TEVFTCK----------------------------- : 349
bfWnt7b : HQFVKTWQCNCK-FHWCCYVKCNQCSER-TEEYTCK----------------------------- : 347
NvWnt5 : KKEVQATRCRCK-FHWCCKVKCKTCIKN-VTTHICN----------------------------- : 358
bfWnt5 : FKREKVERCNCK-FHWCCYVKCKRCRSI-KNVYVCK----------------------------- : 370
mmWnt5a : FKTVQTERCHCK-FHWCCYVKCKKCTEI-VDQFVCK----------------------------- : 360
mmWnt5b : FKSVQVERCHCR-FHWCCFVRCKKCTEV-VDQYVCK----------------------------- : 359
Nvwnt-A : FKLSNQVKCNCE-FIWCCKVQCDMCKKD-WTEYRCR----------------------------- : 365
pdWnta : TVREIKEDCNCK-FHWCCRVECDKCSKK-IEEHFCN----------------------------- : 270
pvWnta : ----------------------------------------------------------------- : -
NvWnt4 : RTEVRQEQCACK-FFWCCHVRCQTCMRR-VEVSYCK----------------------------- : 354
bfWnt4 : HTREVVERCSCK-FHWCCYVKCKTCRRT-VEVHTCK----------------------------- : 353
mmWnt4 : AQVELAERCGCR-FHWCCFVKCRQCQRL-VEMHTCR----------------------------- : 351
NvWnt1 : SSQERVENCNCRVFLGGCEVKCQKCKHE-GSLSNCL----------------------------- : 364
NvWnt6 : TVINEEVNCRCR-FHWCCEVRCKKCKKE-RVVFTCK----------------------------- : 207
bfWnt1 : RQEVTKERCNCT-FQWCCQVKCEECVRT-KTIHTCL----------------------------- : 377
mmWnt1 : RTQRVTERCNCT-FHWCCHVSCRNCTHT-RVLHECL----------------------------- : 370
bfWnt6 : RQVVVGENCKCR-FHWCCVVKCSKCTAV-KTVHECL----------------------------- : 213
mmWnt6 : ESVQLEENCLCR-FHWCCVVQCHRCRVR-KELSLCL----------------------------- : 364
 ● ● ●● ● ● ●
