# Supplementary material for: Wnt and TGF-β Expression in the Sponge Amphimedon queenslandica and the Origin of Metazoan Embryonic Patterning
Source: PLoS One. 2007 Oct 10;2(10):e1031. doi: 10.1371/journal.pone.0001031 (PMC2000352; doi:10.1371/journal.pone.0001031)
Supplement: Figure S3 — (0.03 MB DOC) [file pone.0001031.s004.doc]

**Figure S3. TGF-β phylogenetic analysis**

### 
