# Supplementary material for: Wnt and TGF-β Expression in the Sponge Amphimedon queenslandica and the Origin of Metazoan Embryonic Patterning
Source: PLoS One. 2007 Oct 10;2(10):e1031. doi: 10.1371/journal.pone.0001031 (PMC2000352; doi:10.1371/journal.pone.0001031)
Supplement: Figure S4 — (0.04 MB DOC) [file pone.0001031.s005.doc]

**Figure S4. TGF-β sequence alignment**

* 20 * 40 * 60 * 80 * 100
XactivinD : CCRKDYYVDF--KDIGWNDWIIKPEGYQINYCMGLCPMHIAGAPGMAASFHTTVLNLIKANNIQ---TAVNSCCVPTKRRPLSMLYFDRNNNVLKT-DIA : 94
mminhbc : CCRQEFFVDF--REIGWNDWIIQPEGYAMNFCTGQCPLHVAGMPGISASFHTAVLNLLKANAAAG-TTGRGSCCVPTSRRPLSLLYYDRDSNIVKT-DIP : 96
mmgdf11 : CCRYPLTVDF--EAFGW-DWIIAPKRYKANYCSGQCEYMFM-----QKYPHT---HLVQQANPR---GSAGPCCTPTKMSPINMLYFNDKQQIIYG-KIP : 85
mmgdf8 : CCRYPLTVDF--EAFGW-DWIIAPKRYKANYCSGECEFVFL-----QKYPHT---HLVHQANPR---GSAGPCCTPTKMSPINMLYFNGKEQIIYG-KIP : 85
mmtgfb2 : CCLRPLYIDFK-RDLGW-KWIHEPKGYNANFCAGACPYLWS-----SDTQHTKVLSLYNTINPE---ASASPCCVSQDLEPLTILYYIGNTPKIE--QLS : 88
mmtgfb3 : CCVRPLYIDFR-QDLGW-KWVHEPKGYYANFCSGPCPYLRS-----ADTTHSTVLGLYNTLNPE---ASASPCCVPQDLEPLTILYYVGRTPKVE--QLS : 88
mmtgfB1 : CCVRQLYIDFR-KDLGW-KWIHEPKGYHANFCLGPCPYIWS-----LDTQYSKVLALYNQHNPG---ASASPCCVPQALEPLPIVYYVGRKPKVE--QLS : 88
mmbmp5 : CKKHELYVSF--RDLGWQDWIIAPEGYAAFYCDGECSFPLN--AHMNATNHAIVQTLVHLMFP---DHVPKPCCAPTKLNAISVLYFDDSSNVILK-KYR : 92
mmbmp6 : CKKHELYVSF--QDLGWQDWIIAPKGYAANYCDGECSFPLN--AHMNATNHAIVQTLVHLMNP---EYVPKPCCAPTKLNAISVLYFDDNSNVILK-KYR : 92
NvBMP5-8 : CQRQALHVSF--RKLRWQDWVIAPEGYSAFYCSGECSFPLN--ANMNATNHAIVQTLVHLMNP---KTVPKPCCAPTELSPISVLYFDQDNNVVLK-KYN : 92
XVg1 : CKKRRLYVEF--KDVGWQNWVIAPQGYMANYCYGECPYPLT--EILNGSNHAILQTLVHSIEP---EDIPLPCCVPTKMSPISMLFYDNNDNVVLR-HYE : 92
mmgdf3 : CHRHQLFINF--QDLGWHKWVIAPKGFMANYCHGECPFSMT--TYLNSSNYAFMQALMHMADP----KVPKAVCVPTKLSPISMLYQDSDKNVILR-HYE : 91
mmbmp2 : CKRHPLYVDF--SDVGWNDWIVAPPGYHAFYCHGECPFPLA--DHLNSTNHAIVQTLVNSVN----SKIPKACCVPTELSAISMLYLDENEKVVLK-NYQ : 91
mmbmp4 : CRRHSLYVDF--SDVGWNDWIVAPPGYQAFYCHGDCPFPLA--DHLNSTNHAIVQTLVNSVN----SSIPKACCVPTELSAISMLYLDEYDKVVLK-NYQ : 91
Amdpp : CQRHPLYVDF--SEVGWNDWIVAPPGYQGFYCKGECPFPIA--DHLNTTNHAIVQTLMNSVNP---NNVPPACCVPTTLEAISMLFMNEHSKVVLK-NYQ : 92
NvDpp : CRRHPLYVDF--TDVGWNDWIVAPPGYHAFYCTGVCPYPIA--KHLNATNHAIVQTIMNTVD----SNVPNACCIPTTLNPISILSLNEFDKVVLK-NYK : 91
Dorsalin-1 : CRRTSLHVNF--KEIGWDSWIIAPKDYEAFECKGGCFFPLT--DNVTPTKHAIVQTLVHLQNP---KKASKACCVPTKLDAISILYKDDAGVPTLIYNYE : 93
mmgdf2 : CQKTSLRVNF--EDIGWDSWIIAPKEYDAYECKGGCFFPLA--DDVTPTKHAIVQTLVHLKFP---TKVGKACCVPTKLSPISILYKDDMGVPTLKYHYE : 93
mmbmp10 : CKKTPLYIDF--KEIGWDSWIIAPPGYEAYECRGVCNYPLA--EHLTPTKHAIIQALVHLKNS---QKASKACCVPTKLDPISILYLD-KGVVTYKFKYE : 92
mmgdf5 : CSRKALHVNF--KDMGWDDWIIAPLEYEAFHCEGLCEFPLR--SHLEPTNHAVIQTLMNSMDP---ESTPPTCCVPTRLSPISILFIDSANNVVYK-QYE : 92
mmgdf6 : CSRKPLHVNF--KELGWDDWIIAPLEYEAYHCEGVCDFPLR--SHLEPTNHAIIQTLMNSMDP---GSTPPSCCVPTKLTPISILYIDAGNNVVYK-QYE : 92
mmgdf7 : CSRKSLHVDF--KELGWDDWIIAPLDYEAYHCEGVCDFPLR--SHLEPTNHAIIQTLLNSMAP---DAAPASCCVPARLSPISILYIDAANNVVYK-QYE : 92
DrADMP : CQRQPLYVDF--EEIGWSGWIVSPKGYNAYHCKGSCIFPLS--QNMRPTNHAIVQSIINTLKLN--KGIQTPCCVPDKLYSISLLYFDDDENVVLK-QYD : 93
XlADMP : CQRHPLYVDF--EEIGWSGWIISPRGYNAYHCKGSCPFPLG--QNMRPTNHATVQSIINALKLT--KGVSSPCCVPDKLFSINLLYFDDDENVVLK-QYD : 93
mmbmp3 : CARRYLKVDF--ADIGWSEWIISPKSFDAFYCSGACQFPMP--KSLKPSNHATIQSIVRAVGVV--SGIPEPCCVPEKMSSLSILFFDENKNVVLK-VYP : 93
mmgdf10 : CSRRYLKVDF--ADIGWNEWIISPKSFDAYYCAGACEFPMP--KIVRPSNHATIQSIVRAVGIV--PGIPEPCCVPDKMNSLGVLFLDENRNAVLK-VYP : 93
mmnodal : CRRVKFQVDF--NLIGWGSWIIYPKQYNAYRCEGECPNPVG--EEFHPTNHAYIQSLLKRYQP---HRVPSTCCAPVKTKPLSMLYVD-NGRVLLE-HHK : 91
amphinodal : CKKVEFWVDF--DHIGWGTWIIYPKRFNAFRCEGVCPTPVD--QLYHPTSHAVMTSILNLHKP---GKAPMPCCIPTKLKALSMLYLE-HGEVVLR-HHE : 91
mmgdf9 : CELHDFRLSF--SQLKWDNWIVAPHRYNPRYCKGDCPRAVR--HRYGSPVHTMVQNIIYEKLDP---SVPRPSCVPGKYSPLSVLTIEPDGSIAYK-EYE : 92
mmbmp15 : CSLHPYKVSF--HQLGWDHWIIAPRLYTPNYCKGICTRVLP--YGLNSPNHAIIQSLVNELVNH---SVPQPSCVPYNFLPMSILLIETNGSILYK-EYE : 92
mmgdf15 : CHLETVQATL--EDLGWSDWVLSPRQLQLSMCVGECPHLYR-----SANTHAQIKARLHGLQPD---KVPAPCCVPSSYTPVVLMHRTDSGVSLQT--YD : 88
mmartn : CRLRSQLVPV--SALGLGHSSDELIRFR--FCSGSC-RRARS---QHDLSLASLLGAGALRSPPGSRPISQPCCRPTRYE--AVSFMDVNSTWRT---VD : 87
mmpspn : CRLWSLTLPV--AELGLGYASEEKVIFR--YCAGSCPQEAHT---QHSLVLARLRGRG--------RAHGRPCCQPTSYA--DVTFLDDQHHWQQ---LP : 80
mmnrtn : CGLRELEVRV--SELGLGYTSDETVLFR--YCAGAC-EAAIR---IYDLGLRRLRQRRRVRR---ERARAHPCCRPTAYED-EVSFLDVHSRYHT---LQ : 85
mmgdnf : CVLTAIHLNV--TDLGLGYETKEELIFR--YCSGSC-ESAET---MYDKILKNLSRSRRLTS----DKVGQACCRPVAFDD-DLSFLDDNLVYHI---LR : 84
AmqTgfB : CSKSSLSIDKGQLAQILDIEIDFPETFDLNVCGGHCPGSKYINQFHSKITYLLLATSEVEHLAN--KHHYSKTCVPTKYHSLNYIKFDQNGSVIKT--LD : 96
 ● ● ● ○●

*
XactivinD : DMIVEACGCS : 104
mminhbc : DMVVEACGCS : 106
mmgdf11 : GMVVDRCGCS : 95
mmgdf8 : AMVVDRCGCS : 95
mmtgfb2 : NMIVKSCKCS : 98
mmtgfb3 : NMVVKSCKCS : 98
mmtgfB1 : NMIVRSCKCS : 98
mmbmp5 : NMVVRSCGCH : 102
mmbmp6 : NMVVRACGCH : 102
NvBMP5-8 : KMVVKACGCH : 102
XVg1 : NMAVDECGCR : 102
mmgdf3 : DMVVDECGCG : 101
mmbmp2 : DMVVEGCGCR : 101
mmbmp4 : EMVVEGCGCR : 101
Amdpp : DMVVDGCGCR : 102
NvDpp : DMVIEGCGCR : 101
Dorsalin-1 : GMKVAECGCR : 103
mmgdf2 : GMSVAECGCR : 103
mmbmp10 : GMAVSECGCR : 102
mmgdf5 : DMVVESCGCR : 102
mmgdf6 : DMVVESCGCR : 102
mmgdf7 : DMVVEACGCR : 102
DrADMP : DMVAGSCGCR : 103
XlADMP : DMVAGSCGCH : 103
mmbmp3 : NMTVDSCACR : 103
mmgdf10 : NMSVETCACR : 103
mmnodal : DMIVEECGCL : 101
amphinodal : DMIVDECGCQ : 101
mmgdf9 : DMIATRCTCR : 102
mmbmp15 : GMIAQSCTCR : 102
mmgdf15 : DLVARGCHCA : 98
mmartn : HLSATACGCL : 97
mmpspn : QLSAAACGCG : 90
mmnrtn : ELSARECACV : 95
mmgdnf : KHSAKRCGCI : 94
 ● ●
