# Supplementary material for: Wnt and TGF-β Expression in the Sponge Amphimedon queenslandica and the Origin of Metazoan Embryonic Patterning
Source: PLoS One. 2007 Oct 10;2(10):e1031. doi: 10.1371/journal.pone.0001031 (PMC2000352; doi:10.1371/journal.pone.0001031)
Supplement: Figure S5 — (0.04 MB DOC) [file pone.0001031.s006.doc]

**Figure S5. Alignment of sponge and mouse TGF-β**

* 20 * 40 * 60 * 80 * 100
mmbmp2 : MVAGTRCLLVLLLPQVLLGGAAGLIPELGRKKFAAASSRPLSRPSEDVLSEFELRLLSMFGLKQRPTPSKDVVVPPYMLDLYRRHSGQPGAPAPDHRLER : 100
AmqTgfB : MFTSSVNFFLLLLSLATLTMTFPILEKQQ-------------------LSDAEPHRDVVLRESGDPQPR----LKPYVLSLFESFAANAKEQPDRHRY-- : 75

 * 120 * 140 * 160 * 180 * 200
mmbmp2 : AASRANTVRSFHHEEAVEELPEMSGKTARRFFFNLSSVPSDEFLTSAELQIFR--------EQIQEALGNSSFQHRINIYEIIKPAAANLKFPVTRLLDT : 192
AmqTgfB : -----NTLRSFEITKAQGCK-----DSKYTFTFNMSSIPKEEDISKAIFRVYVNISTSFSSSQGSAQLTLTSNNSNINKKNITTNESQFVDFPVQLHIHD : 165

 * 220 * 240 * 260 * 280 * 300
mmbmp2 : RLVNQNTSQWESFDVTPAVMRWTTQGHTNHGFVVEVAHLEENPGVSKRHVRISRSLHQDEHSWSQIRPLLVTFGHDGKGHPLHKREKRQAKHKQRKRLKS : 292
AmqTgfB : WLKNGQLKYLVNLGITIEANGPLECSSKDMGIVFDDANDSTQP-----TLVVYSFDHDEDRLLEKLNKAISEKMNSSESMADADRTRRSTGKTVSKGDSE : 260
 ▲
 * 320 * 340 * 360 * 380 * 400
mmbmp2 : SCKRHPLYVDFSDVG--WNDWIVAPPGYHAFYCHGECPFP--LADHLNSTNHAIVQT--LVNSVNSKIPKACCVPTELSAISMLYLDENEKVVLKNYQDM : 386
AmqTgfB : ECSKSSLSIDKGQLAQILDIEIDFPETFDLNVCGGHCPGSKYINQFHSKITYLLLATSEVEHLANKHHYSKTCVPTKYHSLNYIKFDQNG-SVIKTLDQF : 359
 ● ● ● ●
 *
mmbmp2 : VVEGCGCR----- : 394
AmqTgfB : SVAECSCVYSYSD : 372
 ● ●
